# Long-Term Neurodevelopmental Outcomes and Prognostic Factors in Neonates with Hypoxic–Ischemic Encephalopathy

**DOI:** 10.3390/jcm15062414

**Published:** 2026-03-21

**Authors:** Ramazan Keçeci, Melek Büyükeren, Fatma Hilal Yılmaz, Beyza Özcan, Ümmügülsüm Pamukçu, Şambaz Yılmaz, Halil Çelik, Ümmügülsüm Esenkaya

**Affiliations:** 1 Division of Neonatology, Department of Pediatrics, Konya City Hospital, University of Health Sciences, İstiklal, Adana Çevre Yolu Cd. No:135/1, 42020 Konya, Turkey; melekbuyukeren@hotmail.com (M.B.); f.h.yilmaz@hotmail.com (F.H.Y.); drbeyzaozcan@gmail.com (B.Ö.); 2 Department of Pediatrics, Konya City Hospital, University of Health Sciences, 42020 Konya, Turkey; glsmummu@gmail.com (Ü.P.); sambazyilmaz@gmail.com (Ş.Y.); 3 Division of Pediatric Neurology, Department of Pediatrics, Konya City Hospital, University of Health Sciences, 42020 Konya, Turkey; celikdrh@gmail.com; 4 Clinic of Obstetrics and Gynecology, Konya City Hospital, University of Health Sciences, 42020 Konya, Turkey; ummugulsumesenkaya@gmail.com

**Keywords:** hypoxic–ischemic encephalopathy, therapeutic hypothermia, neurodevelopmental outcome, prognostic factors, neonate

## Abstract

**Background:** Hypoxic–ischemic encephalopathy (HIE) remains a major cause of neonatal mortality and long-term neurodevelopmental impairment despite advances in perinatal care and the widespread use of therapeutic hypothermia. Reliable early prognostic markers are essential for risk stratification and long-term follow-up planning. This study aimed to evaluate long-term neurodevelopmental outcomes and associated prognostic factors in neonates with HIE treated in the era of therapeutic hypothermia. **Methods:** This retrospective cohort study was conducted in a tertiary neonatal intensive care unit between January 2020 and June 2024. Neonates with gestational age ≥ 35 weeks diagnosed with HIE were included. Clinical characteristics, laboratory parameters, neurophysiological findings, neuroimaging results, and indicators of multiorgan dysfunction were recorded. Long-term neurodevelopmental outcomes were assessed at 18 to 24 months of age. The primary outcome was death or severe neurodevelopmental impairment. Multivariable logistic regression analysis was performed to identify independent predictors of adverse outcomes. **Results:** A total of 99 neonates were included. Therapeutic hypothermia was administered to 86 (86.9%) infants. Severe neurodevelopmental impairment or death occurred in 18 (18.2%) patients. Cerebral palsy was diagnosed in 19 (20.9%) survivors, developmental delay in 12 (13.2%), epilepsy in 16 (17.6%), and feeding difficulties in 9 (9.9%). In multivariable analysis, higher lactate levels (adjusted OR = 1.239, 95% CI = 1.052–1.458), lower Apgar score at 5 min (adjusted OR = 0.570, 95% CI = 0.344–0.944), and renal dysfunction (adjusted OR = 7.947, 95% CI = 2.027–31.164) were independently associated with severe neurodevelopmental impairment or death. Multiorgan dysfunction and abnormal neurophysiological and neuroimaging findings were significantly associated with adverse outcomes. **Conclusions:** Early biochemical markers, neurological assessments, neurophysiological recordings, neuroimaging patterns, and systemic organ dysfunction are closely associated with long-term neurodevelopmental outcomes in neonates with HIE. A multidimensional approach to early prognostic evaluation may improve risk stratification and guide targeted follow-up and intervention strategies.

## 1. Introduction

HIE continues to be a primary cause of death and prolonged neurological impairment in neonates. HIE is a primary etiological factor contributing to irreversible central nervous system damage, which can lead to cerebral palsy, epilepsy, and enduring neurodevelopmental deficits. It is the most clearly characterized clinical subtype of neonatal encephalopathy (NE) induced by antenatal, intrapartum, or postnatal occurrences resulting in perinatal asphyxia [1,2,3].

The incidence of HIE is reported to be between 1.5 and 3 per 1000 live births in high-income nations, although significantly elevated rates have been seen in low- and middle-income regions, where access to optimum perinatal care is restricted. A recent population-based research from New Zealand indicated a prevalence of newborn encephalopathy of 1.2 per 1000 live births, which is lower than estimates from prior assessments published in 2010. However, significant diversity is seen in population-based and hospital-based research, with reported incidence rates for HIE varying from 1 to 8 per 1000 live births [4]. This variability has been ascribed to discrepancies in diagnostic criteria, case definitions, and variances in prenatal and neonatal care procedures among facilities.

Data from Türkiye further reveal that HIE remains a significant clinical burden in neonatal intensive care units (NICUs). A multicenter research published in 2008 by the Turkish Neonatal Society Hypoxic–Ischemic Encephalopathy research Group identified 93 cases of HIE among 19,857 live-born babies, resulting in an incidence rate of 2.6 per 1000 live births and representing roughly 1.2% of NICU hospitalizations [4]. Nevertheless, these data primarily reflect the pre-therapeutic hypothermia era and may not accurately reflect current clinical outcomes.

Therapeutic hypothermia (TH) is presently recognized as the standard treatment for neonates born at or after 36 weeks of gestation with moderate to severe HIE. Randomized controlled studies and meta-analyses have shown that TH significantly decreases mortality and the likelihood of cerebral palsy, sensory deficits, and prolonged neurodevelopmental delays [3,4,5]. However, it is possible that the efficacy and safety of TH may not be consistent across all therapeutic contexts and over all patient types. Previous studies conducted in low- and middle-income countries have reported that therapeutic hypothermia may not provide the same benefits observed in high-income settings and may even be associated with increased adverse events in certain contexts, possibly due to differences in perinatal care, infection burden, and supportive intensive care resources [6].

Moreover, there is little information to distinctly ascertain the advantages or possible detriments of TH in infants diagnosed with mild HIE. Recent observational data indicate that newborns with moderate hypoxic–ischemic encephalopathy (HIE) may not be as neurologically benign as previously believed, with a few experiencing negative neurodevelopmental consequences in later childhood. These findings emphasize the necessity of assessing HIE not just based on acute neurological severity but also as a systemic condition marked by diverse levels of multiorgan involvement [7,8].

Accurate early prognostication remains challenging, and multiple clinical, biochemical, neurophysiological, and imaging parameters have been investigated as potential predictors of long-term outcome. Although several prognostic factors have been identified, few studies have comprehensively evaluated biochemical markers, neurophysiological findings, neuroimaging patterns, and multiorgan dysfunction simultaneously as predictors of long-term outcomes in the therapeutic hypothermia era [9,10,11,12]. Therefore, the present study aimed to retrospectively evaluate neonatal clinical characteristics, multiorgan dysfunction, EEG, and MRI findings in neonates diagnosed with HIE and admitted to the NICU, and to investigate their associations with long-term neurodevelopmental outcomes. By providing contemporary data from a tertiary care center in Türkiye, this study seeks to contribute to the identification of clinically relevant prognostic markers that may improve risk stratification and long-term follow-up strategies in infants affected by HIE.

## 2. Methods

### 2.1. Study Design and Setting

This study was designed as a single-center retrospective cohort study conducted in the NICU of Konya City Hospital. Medical records of neonates diagnosed with HIE and admitted between January 2020 and June 2024 were reviewed retrospectively.

The NICU is a tertiary referral center providing comprehensive neonatal intensive care, including therapeutic hypothermia for eligible infants with moderate to severe HIE.

### 2.2. Ethical Approval

The study protocol received permission from the Konya City Hospital Ethics Committee (approval number: 2025-201; date: 15 September 2025). The study protocol was reviewed and approved by the institutional ethics committee, which granted retrospective approval for the analysis of existing clinical data and medical records. Informed consent was waived due to the study’s retrospective design. All methods adhered to the principles of the Declaration of Helsinki, and patient confidentiality was rigorously upheld.

### 2.3. Study Population

All consecutive neonates diagnosed with hypoxic–ischemic encephalopathy and admitted to the neonatal intensive care unit between January 2020 and June 2024 were screened for eligibility. Infants were eligible for inclusion if they met all of the following criteria:

- Gestational age ≥ 35 weeks;
- Admission to the NICU within the first hours of life;
- Diagnosis of hypoxic–ischemic encephalopathy based on clinical findings and perinatal history;
- Availability of sufficient clinical data to classify HIE severity and short-term outcomes.

Infants were excluded if they had:

- Major congenital anomalies or chromosomal abnormalities;
- Congenital central nervous system malformations;
- Congenital infections;
- Inborn errors of metabolism or alternative causes of neonatal encephalopathy;
- Incomplete medical records precluding outcome assessment.

### 2.4. Clinical Assessment and HIE Classification

HIE severity was classified according to the Sarnat and Sarnat staging system as mild (stage I), moderate (stage II), or severe (stage III), based on neurological examination findings during the first days of life.

Perinatal and neonatal variables collected included gestational age, maternal age, birth weight, sex, mode of delivery, Apgar scores at 5 and 10 min, need for resuscitation, and initial blood gas parameters (pH, base excess, lactate).

### 2.5. TH and NICU Management

Therapeutic hypothermia was administered to infants with moderate or severe HIE according to international guidelines. Although international guidelines generally recommend therapeutic hypothermia for infants ≥ 36 weeks of gestation, infants with gestational age ≥ 35 weeks were included in the present study in accordance with the local NICU protocol. Eligibility criteria included evidence of perinatal asphyxia (cord or early postnatal pH ≤ 7.0 or base deficit ≥ 16 mmol/L, low Apgar scores, or need for prolonged resuscitation) together with moderate or severe encephalopathy on neurological examination In selected cases with mild HIE showing early neurological deterioration or abnormal aEEG abnormalities within the first hours of life, therapeutic hypothermia was initiated based on clinical judgment at the discretion of the attending neonatologist. Cooling was initiated as early as possible within the first 6 h of life and maintained for 72 h, followed by controlled rewarming.

Details regarding the timing of hypothermia initiation, duration, complications during cooling, need for mechanical ventilation, inotropic support, antiseizure medications, and other supportive treatments were recorded.

### 2.6. EEG and Neuroimaging

Neurophysiological monitoring involved both amplitude-integrated EEG (aEEG) and conventional EEG. Recordings using aEEG were conducted early while the patient was undergoing postnatal therapeutic hypothermia, followed by conventional EEG monitoring. The neonatal EEG was interpreted based on standardized criteria for assessing background activity and detecting seizures. Seizures were classified primarily using electrographic criteria. aEEG background activity was categorized as: normal, mildly to moderately abnormal, or severely abnormal (including burst suppression, continuous low-voltage activity, or isoelectric patterns). Electrographic seizures were recorded as present or absent.

Brain MRI, when available, was performed during the neonatal period according to clinical practice. MRI findings were classified based on injury patterns (normal, watershed injury, basal ganglia/thalamic involvement, or diffuse injury). Diffuse injury was defined as extensive involvement of both cortical and deep gray matter structures accompanied by widespread white matter abnormalities. When multiple injury patterns were present, classification was based on the predominant injury pattern.

### 2.7. Definition of Organ Dysfunction

Organ dysfunction was assessed retrospectively based on clinical findings, laboratory results, and treatment requirements documented during NICU stay. Each organ system was evaluated independently.

Cardiovascular dysfunction was defined as persistent hypotension requiring inotropic or vasopressor support for ≥24 h.

Respiratory dysfunction was defined as the requirement for invasive mechanical ventilation or significant respiratory support with high oxygen supplementation.

Renal dysfunction was defined as oliguria or anuria (<0.5 mL/kg/hour for ≥24 h), elevated serum creatinine above gestational age-adjusted reference values, or a documented diagnosis of acute kidney injury.

Hepatic dysfunction was defined as transaminase levels (AST or ALT) ≥ 2 times the upper limit of normal and/or impaired synthetic function indicated by INR > 1.5.

Hematologic/coagulation dysfunction was defined as thrombocytopenia (<100,000/mm^3^), clinically significant coagulopathy, or requirement for blood product transfusion.

Metabolic dysfunction was defined as recurrent hypoglycemia (<45 mg/dL) or clinically significant electrolyte disturbances requiring treatment.

### 2.8. Long-Term Neurodevelopmental Assessment

Long-term neurodevelopmental outcomes were evaluated using a structured, clinically based assessment approach, as standardized developmental tests were not routinely available for all patients. Neurodevelopmental status was determined from pediatric neurology evaluations, developmental clinic records, physiotherapy reports, and documented functional milestones. Follow-up assessments were performed at ≥18–24 months of age, whenever available.

Primary outcome:

The primary composite outcome was defined as death at any time before follow-up or severe neurodevelopmental impairment (sNDI). sNDI was diagnosed if at least one of the following was present:

- Cerebral palsy with significant motor impairment corresponding to Gross Motor Function Classification System (GMFCS) levels II–V;
- Severe global developmental delay requiring special education or resulting in functional dependence;
- Epilepsy requiring ongoing antiseizure medication, particularly polytherapy;
- Severe hearing impairment requiring hearing aids or cochlear implantation;
- Severe visual impairment, including cortical visual impairment or functional blindness.

Secondary outcomes:

- Secondary neurodevelopmental outcomes included cerebral palsy, gross motor function (independent walking, assisted walking, non-ambulatory), developmental level (age-appropriate, mild–moderate delay, severe delay), epilepsy, feeding status, hearing and visual impairment. Feeding impairment was defined as the requirement for tube feeding (nasogastric or gastrostomy feeding) at follow-up.

### 2.9. Statistical Analysis

The data were analyzed utilizing the IBM SPSS Statistics Standard Concurrent User Version 30 (IBM Corp., Armonk, NY, USA) statistical software tool. Descriptive data included the number of units (*n*), percentage (%), mean ± standard deviation, median, interquartile range, minimum, and maximum values. The Shapiro–Wilk normality test was employed to assess the normality of the data. The variance homogeneity of the groups was assessed using the Levene test. Intergroup comparisons for numerical variables were conducted using the independent samples *t*-test for normally distributed data and the Mann–Whitney U test for non-normally distributed data. One-way ANOVA was employed for comparing more than two groups of numerical variables when the data had a normal distribution; however, the Kruskal–Wallis test was utilized when the data lacked normality. The Duncan test was employed as a multiple comparison test in one-way ANOVA, whereas the Dunn–Bonferroni test was utilized in the Kruskal–Wallis analysis. Chi-square tests (Fisher–Freeman–Halton exact test, Yates chi-square, Fisher exact test) were employed to compare groups with variables. Upon finding significant findings from the chi-square analysis, subgroup analyses were performed utilizing the Bonferroni-adjusted two-proportion Z test. The primary and secondary outcomes were assessed by binary logistic regression analysis. In the preliminary model, factors exhibiting *p* < 0.10 in univariate analyses were incorporated. The final model was attained using the backward elimination Wald approach. A *p* < 0.05 value was deemed statistically significant. The selection of variables for multivariable regression models was determined by their clinical relevance and univariate statistical significance. In order to mitigate the risk of overfitting, the models were limited in terms of the number of predictors and the number of outcome events.

## 3. Results

### 3.1. Study Population and Baseline Characteristics

**Figure 1:**
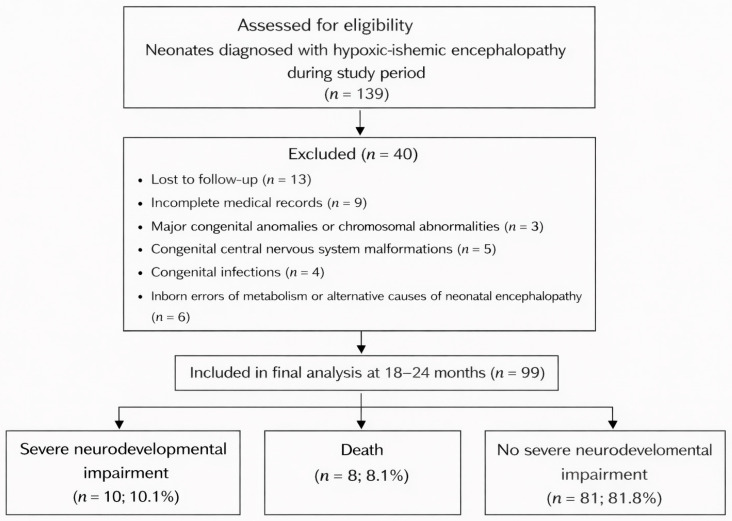
Flow diagram showing patient selection, exclusions, and final study population.

During the study period, a total of 139 neonates diagnosed with HIE were screened for eligibility. After exclusion of infants with incomplete medical records, loss to follow-up, congenital anomalies, congenital infections, or alternative causes of neonatal encephalopathy, 99 infants were included in the final analysis. The patient selection process is illustrated in Figure 1.

Most infants were born at term and received therapeutic hypothermia according to standard clinical protocols. Moderate and severe encephalopathy constituted the majority of cases, and abnormal neurophysiological and neuroimaging findings were observed in a substantial proportion of patients. Multiorgan dysfunction was common, particularly involving respiratory, cardiovascular, renal, and metabolic systems. Detailed baseline clinical, neurophysiological, neuroimaging, and systemic characteristics are presented in Table 1.

### 3.2. Primary Outcome

The primary outcome of the study was the composite outcome of sNDI or death during follow-up.

At 18–24 months of age, sNDI was identified in 10 infants (10.1%), while 8 infants (8.1%) died during follow-up. Overall, the composite primary outcome occurred in 18 infants. Detailed neurodevelopmental outcomes are presented in Table 1.

### 3.3. Secondary Outcomes

Secondary outcomes included cerebral palsy, motor impairment, developmental delay, epilepsy, sensory impairment, and feeding impairment.

Among surviving infants, cerebral palsy was diagnosed in 19 infants (20.9%). Independent walking was achieved by 76 children (83.5%), whereas 9 (9.9%) required assisted walking and 6 (6.6%) were unable to walk. Developmental delay was identified in 12 infants, including 6 with mild-to-moderate delay and 6 with severe delay. Epilepsy was diagnosed in 16 infants (17.6%). Feeding impairment requiring tube feeding was observed in nine infants (9.9%), while the majority of survivors achieved full oral feeding (Table 1).

### 3.4. Neurophysiological and Neuroimaging Findings

Clinical, biochemical, neurophysiological, and neuroimaging variables were compared according to neurodevelopmental outcome groups (sNDI absent, sNDI present, and death), as shown in Table 2. Infants with adverse outcomes had significantly lower cord blood pH, higher base excess values, and markedly elevated lactate levels compared with infants without sNDI (*p* < 0.05). Lower Apgar scores at both 5 and 10 min were also significantly associated with worse outcomes.

Severe HIE stage, abnormal aEEG background patterns, electrographic seizures, and pathological MRI injury patterns were significantly more frequent among infants with sNDI or death (all *p* < 0.001). In addition, the presence of any organ dysfunction and specific organ system involvement, including cardiovascular, respiratory, renal, hepatic, hematological, and metabolic dysfunction, was strongly associated with adverse outcomes (Table 2).

### 3.5. Univariate Analysis of Predictors of Adverse Outcomes

Markers reflecting the severity of perinatal hypoxic–ischemic insult were strongly associated with adverse outcomes. Biochemical indicators of metabolic derangement, including cord blood acidosis and elevated lactate levels, showed significant associations with multiple adverse neurodevelopmental endpoints. Similarly, lower Apgar scores and higher encephalopathy severity were consistently associated with worse outcomes.

Neurological assessment tools demonstrated strong prognostic value. Abnormal aEEG background activity, electrographic seizures on EEG, and pathological MRI injury patterns were significantly associated with all major adverse outcomes.

Systemic involvement was also an important determinant of prognosis. Multiorgan dysfunction, including cardiovascular, respiratory, renal, and metabolic impairment, was significantly associated with adverse neurodevelopmental outcomes. Prolonged hospitalization was also associated with worse outcomes. Detailed univariate associations are presented in Table 3.

### 3.6. Multivariate Logistic Regression Analysis

Multivariate logistic regression analysis identified several independent predictors of adverse outcomes (Table 4).

Lactate level emerged as the only variable consistently associated with multiple adverse neurodevelopmental outcomes, including severe neurodevelopmental impairment or death, cerebral palsy, motor impairment, developmental delay, and epilepsy.

Lower Apgar scores were independently associated with increased risk of adverse neurological outcomes, particularly severe neurodevelopmental impairment, developmental delay, and epilepsy.

Organ dysfunction was also a strong independent predictor of adverse outcomes. Renal dysfunction was independently associated with severe neurodevelopmental impairment or death, cardiovascular dysfunction predicted motor impairment, and overall organ dysfunction predicted cerebral palsy. In addition, metabolic dysfunction was identified as a strong independent predictor of feeding impairment.

Overall, these findings indicate that early biochemical markers, neurological assessments, and systemic organ dysfunction are strongly associated with long-term neurodevelopmental outcomes in infants with hypoxic–ischemic encephalopathy.

## 4. Discussion

In this retrospective cohort study, we evaluated long-term neurodevelopmental outcomes and associated prognostic factors in neonates with HIE treated in a tertiary neonatal intensive care unit in the era of therapeutic hypothermia. HİE remains a major cause of neonatal mortality and long-term neurodevelopmental impairment despite advances in therapeutic strategies such as therapeutic hypothermia [3]. Our findings demonstrate that early markers of perinatal asphyxia severity, neurophysiological and neuroimaging abnormalities, and multiorgan dysfunction are closely associated with adverse long-term outcomes.

One of the most notable findings of our study was that lactate level emerged as the only variable independently associated with multiple adverse neurodevelopmental outcomes, including severe neurodevelopmental impairment or death, cerebral palsy, developmental delay, epilepsy, and motor impairment. Elevated lactate levels reflect the severity of systemic hypoxia and impaired oxidative metabolism during hypoxic–ischemic injury. Early metabolic biomarkers have been increasingly investigated as indicators of the severity of hypoxic–ischemic injury and predictors of neurodevelopmental outcomes [13,14]. Our findings are consistent with previous studies indicating that early metabolic derangements reflect the severity of hypoxic–ischemic injury and predict long-term neurological morbidity [15,16,17]. Persistent metabolic acidosis and hyperlactatemia have been shown to correlate with neuronal injury and unfavorable neurodevelopmental trajectories [18]. Our study extends these observations by demonstrating that lactate is independently associated with multiple long-term neurodevelopmental outcomes.

Furthermore, organ failure has surfaced as a significant predictor of unfavorable neurological and motor outcomes. Increasing evidence suggests that HIE is a multisystem disorder, and the extent of systemic involvement reflects the severity of global hypoxic–ischemic insult [2,19,20]. Previous studies have reported that early organ dysfunction is associated with higher mortality and worse neurodevelopmental outcomes [4,21]. Our findings extend these observations by demonstrating their independent association with long-term morbidity.

Neurophysiological monitoring using amplitude-integrated EEG and conventional EEG has been widely recognized as a reliable predictor of outcome in neonates with HIE. Severely abnormal background patterns and electrographic seizures have consistently been associated with poor neurodevelopmental prognosis [22,23]. Similarly, neonatal brain MRI, particularly injury patterns involving the basal ganglia and thalami or diffuse cortical injury, has been shown to correlate strongly with long-term motor and cognitive impairment [24,25,26]. Our findings are in agreement with these reports and further support the complementary role of neurophysiological and neuroimaging assessments.

Despite the widespread implementation of therapeutic hypothermia, a substantial proportion of infants with moderate and severe HIE continue to experience adverse outcomes. Large randomized trials and meta-analyses have demonstrated that therapeutic hypothermia reduces mortality and severe disability but does not eliminate neurological morbidity [27,28,29]. Contemporary registry data indicate that approximately 20% to 40% of cooled infants develop significant neurodevelopmental impairment [4,15]. The outcome rates observed in our cohort are comparable to these reports, suggesting that residual neurological morbidity remains an important clinical challenge.

In line with international guidelines and consensus statements, therapeutic hypothermia was applied to eligible infants within the recommended therapeutic window [30,31]. However, our data indicate that hypothermia alone may not fully mitigate the effects of severe hypoxic–ischemic injury, particularly in infants with extensive systemic involvement. This observation is consistent with recent guideline updates emphasizing the importance of comprehensive supportive care and close neurological monitoring in addition to temperature management.

Feeding impairment was observed in a subset of survivors and was closely related to markers of severe perinatal compromise and advanced HIE stage. Although data on long-term feeding outcomes in HIE remain limited, previous follow-up studies have suggested that oromotor dysfunction and swallowing difficulties may persist beyond infancy, particularly in children with severe neonatal encephalopathy [1,7,25]. Our findings support these observations and indicate that early neurological and systemic injury may have lasting effects on feeding and swallowing function.

The findings of this study have important clinical implications for the management of neonates with hypoxic–ischemic encephalopathy. Early assessment of biochemical markers, neurological status, neurophysiological recordings, neuroimaging patterns, and systemic organ function may facilitate the timely identification of infants at high risk for adverse long-term outcomes. Routine monitoring of lactate levels, careful evaluation of Apgar scores, and systematic assessment of multiorgan dysfunction may provide valuable information beyond traditional neurological examinations.

Integrating these parameters into standardized follow-up protocols may help optimize individualized surveillance and early intervention strategies. Furthermore, the observed association between early neurological injury and later feeding difficulties highlights the importance of multidisciplinary follow-up programs involving neonatologists, pediatric neurologists, developmental specialists, and feeding therapists. Early identification of infants at risk may improve access to supportive therapies and potentially mitigate long-term functional limitations.

This study has several strengths. First, we comprehensively evaluated clinical, biochemical, neurophysiological, and neuroimaging predictors within a single cohort. Second, multiple long-term neurodevelopmental outcomes were assessed, allowing a multidimensional evaluation of prognostic factors.

Several limitations of this study should be acknowledged. First, the retrospective and single-center design may limit the generalizability of our findings. Second, although follow-up was based on structured clinical documentation, standardized developmental assessment tools such as the Bayley Scales were not available for all patients, which may limit the precision of developmental outcome classification. Third, the relatively small number of adverse events for certain outcomes, particularly feeding and sensory impairments, limited the statistical power of multivariable analyses. Fourth, potential residual confounding factors, including socioeconomic status and access to rehabilitation services, could not be fully evaluated. Finally, variations in supportive care practices over time may have influenced outcomes.

Future studies are needed to further validate the prognostic role of early biochemical and systemic markers in neonates with hypoxic–ischemic encephalopathy. Prospective multicenter studies with larger cohorts and standardized neurodevelopmental assessments may help refine prognostic models and improve early risk stratification. In addition, integrating clinical, biochemical, neurophysiological, and neuroimaging parameters into predictive models may enhance the accuracy of outcome prediction and support individualized management strategies in neonatal intensive care units.

## 5. Conclusions

This study demonstrates that early markers of perinatal asphyxia severity, neurophysiological and neuroimaging abnormalities, and multiorgan dysfunction are associated with adverse long-term neurodevelopmental outcomes in neonates with hypoxic–ischemic encephalopathy. Our findings support a multidimensional approach to prognostic assessment that integrates clinical, laboratory, neurological, neurophysiological, neuroimaging, and systemic parameters. Such an approach may facilitate improved risk stratification, targeted follow-up, and timely intervention in this vulnerable population. Further prospective multicenter studies incorporating standardized developmental assessments are warranted to validate these findings and to refine prognostic models in the era of therapeutic hypothermia.

## Figures and Tables

**Table 1:** Perinatal characteristics, early neurological findings, neuroimaging results, and long-term outcomes in neonates with hypoxic–ischemic encephalopathy.

Variable	Value	Variable	Value
Sex, *n* (%)		MRI, *n* (%)	
Female	51 (51.5)	Normal	51 (51.5)
Male	48 (48.5)	Watershed	22 (22.2)
Maternal age	26.56 ± 6.18 (17–42)	Basal ganglia/thalamus Diffuse injury	11 (11.1) 15 (15.2)
Gestational age	38.40 ± 1.71 (35–42)		
Birth weight	3200 (1800–4485)	Age at MRI, days	4 (2–6)
Length	49.26 ± 0.99 (45–51)	Organ dysfunction, *n* (%)	39 (39.4)
Head circumference	35.23 ± 0.74 (34–36)	KVC, *n* (%) Respiratory, *n* (%) Renal, *n* (%) Hepatic, *n* (%) Hematological, *n* (%) Metabolic, *n* (%)	27 (27.3) 35 (35.4) 20 (20.2) 6 (6.1) 6 (6.1) 18 (18.2)
Type of Delivery, *n* (%)			
SVD	59 (59.6)		
C/S	40 (40.4)		
Cord blood gas			
pH	6.93 ± 0.11 (6.60–7.20)		
BE	18.02 ± 4.96 (5.0–31.0)	Length of hospital stay	9 (3–270)
Lactate	13.0 (6.0–25.0)	Neurodevelopmental assessment at 18–24 months, *n* (%)	
Apgar score at 5 min	3 (0–7)	sNDI absent	81 (81.8)
Apgar score at 10 min	5 (1–8)	sNDI present	10 (10.1)
Sarnat, *n* (%)		Death	8 (8.1)
Mild (stage I)	41 (41.4)	Cerebral Palsy, *n* (%) *	19 (20.9)
Moderate (stage II)	45 (45.5)	Motor Function, *n* (%) *	
Severe (stage III)	13 (13.1)	Independent walking	76 (83.5)
Resuscitation in delivery room, *n* (%)		Assisted walking	9 (9.9)
No	19 (19.2)	Inability to walk	6 (6.6)
Yes	80 (80.8)	Developmental delay, *n* (%) *	
TH, *n* (%)		Age-appropriate	79 (86.8)
No	13 (13.1)	Mild–moderate delay	6 (6.6)
Yes	86 (86.9)	Severe delay	6 (6.6)
Timing of TH, hours	1 (1–6)	Epilepsy, *n* (%) *	16 (17.6)
aEEG, *n* (%)		Severe hearing loss, *n* (%) *	3 (3.3)
Normal	78 (78.8)	Visual impairment, *n* (%) *	0 (0.0)
Mild to moderately abnormal	13 (13.1)	Feeding, *n* (%)	
Severely abnormal	8 (8.1)	Tube feeding	9 (9.9)
EEG, *n* (%)		Full oral feeding	82 (90.1)
No electrographic seizures	64 (64.6)		
Electrographic seizures present	35 (35.4)		

sNDI: Severe neurodevelopmental impairment, *n*: Number of patients, %: Percentage value, numerical variables are summarized as mean ± standard deviation (min-max) for normally distributed data, and as median (min-max) for non-normally distributed data. *: Patients with deaths are not included.

**Table 2:** Clinical, biochemical, and neurological characteristics according to long-term neurodevelopmental outcome in neonates with hypoxic–ischemic encephalopathy.

Variables	Neurodevelopmental Assessment at 18–24 Months			Test Statistics	
	sNDI (−) *n* = 81	sNDI (+) *n* = 10	Death *n* = 8	Test Value	*p* Value
Sex, *n* (%)					
Female	42 (51.9)	5 (50.0)	4 (50.0)	0.135	1.000 ^¥^
Male	81 (48.1)	5 (50.0)	4 (50.0)		
Maternal age	26.36 ± 6.35	27.70 ± 5.01	27.13 ± 6.22	0.243	0.785 ^†^
Gestational age	38.47 ± 1.74	38.20 ± 1.48	38.00 ± 1.85	0.347	0.708 ^†^
Birthweight	3200.0 (2690.0–3500.0)	3057.5 (2885.0–3421.3)	3297.5 (2808.8–3467.5)	0.202	0.904 ^&^
Length	49.28 ± 1.00	49.10 ± 0.99	49.25 ± 0.89	0.153	0.858 ^†^
Head circumference	35.21 ± 0.74	35.30 ± 0.82	35.38 ± 0.74	0.224	0.800 ^†^
Type of Delivery, *n* (%)					
SVD	50 (61.7)	5 (50.0)	4 (50.0)	0.999	0.626 ^¥^
C/S Cord blood gas	31 (38.3)	5 (50.0)	4 (50.0)		
pH	6.94 ± 0.11 ^a^	6.85 ± 0.14 ^b^	6.89 ± 0.12 ^ab^	3.709	0.028 ^†^
BE	17.12 ± 4.69 ^a^	22.30 ± 5.25 ^b^	21.75 ± 2.71 ^b^	8.377	<0.001 ^†^
Lactate	12.0 (10.0–15.5) ^a^	20.0 (17.3–22.8) ^b^	18.0 (14.3–21.8) ^b^	17.947	<0.001 ^&^
Apgar score at 5 min	3.0 (3.0–5.0) ^a^	2.0 (0.8–4.0) ^b^	1.5 (0.3–3.0) ^b^	12.779	0.002 ^&^
Apgar score at 10 min	6.0 (4.0–7.0) ^a^	3.5 (3.0–5.0) ^b^	3.0 (3.0–4.8) ^b^	18.396	<0.001 ^&^
Sarnat, *n* (%)					
Stage I	41 (50.6) ^a^	0 (0.0) ^b^	0 (0.0) ^b^		
Stage II	39 (48.1) ^a^	5 (50.0) ^a^	1 (12.5) ^a^	48.513	<0.001 ^¥^
Stage III	1 (1.3) ^a^	5 (50.0) ^b^	7 (87.5) ^b^		
Resuscitation in delivery room, *n* (%)					
No	19 (23.5)	0 (0.0)	0 (0.0)	4.390	0.084 ^¥^
Yes	62 (76.5)	10 (100.0)	8 (100.0)		
TH, *n* (%)					
Not undergone	13 (16.0)	0 (0.0)	0 (0.0)	2.089	0.355 ^¥^
Undergone	68 (84.0)	10 (100.0)	8 (100.0)		
Timing of TH, hours	1.5 (1.0–4.0)	1.0 (1.0–4.5)	1.0 (1.0–4.8)	0.029	0.985 ^&^
aEEG, *n* (%)					
Normal	77 (95.1) ^a^	1 (10.0) ^b^	0 (0.0) ^b^		
Mild to moderately abnormal	4 (4.9) ^a^	8 (80.0) ^b^	1 (12.5) ^a^	74.158	<0.001 ^¥^
Severe abnormal	0 (0.0) ^a^	1 (10.0) ^b^	7 (87.5) ^c^		
EEG, *n* (%)					
No electrographic seizures	64 (79.0) ^a^	0 (0.0) ^b^	0 (0.0) ^b^	39.966	<0.001 ^¥^
Electrographic seizures present	17 (21.0) ^a^	10 (100.0) ^b^	8 (100.0) ^b^		
MRI, *n* (%)					
Normal	51 (63.0) ^a^	0 (0.0) ^b^	0 (0.0) ^b^		
Watershed	21 (25.9) ^a^	1 (10.0) ^a^	0 (0.0) ^a^	59.358	<0.001 ^¥^
Basal ganglia/thalamus	8 (9.9) ^a^	2 (20.0) ^b^	1 (12.5) ^a^		
Diffuse injury	1 (1.2) ^a^	7 (70.0) ^a^	7 (87.5) ^b^		
Age at MRI, days	4.0 (3.0–5.0)	4.5 (3.8–5.3)	3.5 (3.0–5.5)	1.283	0.526 ^&^
Any organ dysfunction *n* (%)					
No	60 (74.1) ^a^	0 (0.0) ^b^	0 (0.0) ^b^	34.638	<0.001 ^¥^
Yes	21 (25.9) ^a^	10 (100.0) ^b^	8 (100.0) ^b^		
KVC, *n* (%)					
No	71 (87.7) ^a^	1 (10.0) ^b^	0 (0.0) ^b^	45.447	<0.001 ^¥^
Yes	10 (12.3) ^a^	9 (90.0) ^b^	8 (100.0) ^b^		
Respiratory, *n* (%)					
No	64 (79.0) ^a^	0 (0.0) ^b^	0 (0.0) ^b^	39.966	<0.001 ^¥^
Yes	17 (21.0) ^a^	10 (100.0) ^b^	8 (100.0) ^b^		
Renal, *n* (%)					
No	72 (88.9) ^a^	5 (50.0) ^b^	2 (25.0) ^b^	20.510	<0.001 ^¥^
Yes	9 (11.1) ^a^	5 (50.0) ^b^	6 (75.0) ^b^		
Hepatic, *n* (%)					
No	79 (97.5) ^a^	9 (90.0) ^ab^	5 (62.5) ^b^	10.726	0.003 ^¥^
Yes	2 (2.5) ^a^	1 (10.0) ^ab^	3 (37.5) ^b^		
Hematological, *n* (%)					
No	79 (97.5) ^a^	8 (80.0) ^b^	6 (75.0) ^b^	9.104	0.009 ^¥^
Yes	2 (2.5) ^a^	2 (20.0) ^b^	2 (25.0) ^b^		
Metabolic, *n* (%)					
No	79 (97.5) ^a^	0 (0.0) ^b^	2 (25.0) ^b^	62.465	<0.001 ^¥^
Yes	2 (2.5) ^a^	10 (100.0) ^b^	6 (75.0) ^b^		
Length of hospital stay	9 (7–13)	44 (21–102)	-	4.178	<0.001 ^§^

*n*: Number of patients, %: Column percentage, numerical variables are summarized as mean ± standard deviation for normally distributed data and median (first quartile–third quartile) for non-normally distributed data. †: One-way analysis of variance, &: Kruskal–Wallis analysis, ¥: Fisher–Freeman–Halton exact test, §: Mann–Whitney U test, a, b, and c superscripts in the same row indicate intergroup differences. Groups with the same superscripts are not statistically different.

**Table 3:** Univariate analysis of clinical and laboratory predictors associated with adverse neurodevelopmental outcomes in neonates with hypoxic–ischemic encephalopathy.

Variable	sNDI or Death *p*	Cerebral Palsy *p*	Motor Impairment *p*	Developmental Delay *p*	Epilepsy *p*	Feeding Impairment *p*
Cord blood pH	0.028	0.034	0.061	0.057	0.044	0.031
Base excess	<0.001	0.001	0.004	0.003	0.008	0.189
Lactate	<0.001	<0.001	<0.001	0.004	0.006	0.118
Apgar score at 5 min	0.002	0.004	0.007	0.007	0.007	0.021
Apgar score at 10 min	<0.001	0.002	0.004	0.003	0.001	0.009
Sarnat stage	<0.001	<0.001	<0.001	<0.001	<0.001	<0.001
Abnormal aEEG	<0.001	<0.001	<0.001	<0.001	<0.001	<0.001
Abnormal EEG	<0.001	<0.001	<0.001	<0.001	<0.001	0.002
Abnormal brain MRI	<0.001	<0.001	<0.001	<0.001	<0.001	<0.001
Any organ dysfunction	<0.001	<0.001	<0.001	<0.001	<0.001	0.007
Cardiovascular dysfunction	<0.001	<0.001	<0.001	<0.001	<0.001	0.002
Respiratory dysfunction	<0.001	<0.001	<0.001	<0.001	<0.001	0.018
Renal dysfunction	<0.001	0.008	0.090	0.118	0.015	1.000
Metabolic dysfunction	<0.001	<0.001	<0.001	<0.001	<0.001	<0.001
Length of hospital stay	<0.001	<0.001	<0.001	<0.001	<0.001	0.003

Univariate analyses were performed to identify clinical and laboratory variables associated with adverse neurodevelopmental outcomes and feeding impairment. Continuous variables were analyzed using independent samples *t*-test, Mann–Whitney U test, one-way ANOVA, or Kruskal–Wallis test as appropriate. Categorical variables were analyzed using chi-square test, Fisher’s exact test, or Fisher–Freeman–Halton test. Severe neurodevelopmental impairment (sNDI) was defined as sNDI or death. Motor impairment was defined as assisted walking or inability to walk. Developmental delay included both mild-to-moderate and severe delay. Feeding impairment was defined as requirement for feeding support at follow-up. Variables with *p* < 0.05 were considered statistically significant and were included in multivariate logistic regression models. Abbreviations: HIE, hypoxic–ischemic encephalopathy; aEEG, amplitude-integrated electroencephalography; EEG, electroencephalography; MRI, magnetic resonance imaging.

**Table 4:** Summary of independent predictors of adverse neurodevelopmental outcomes in neonates with hypoxic–ischemic encephalopathy: Multiple binary logistic regression analyses.

Outcome	Independent Predictor	β	SE	Wald	*p* Value	Odds Ratio	95% CI
Severe neurodevelopmental impairment or death	Lactate	0.214	0.083	6.634	0.010	1.239	1.052–1.458
	Apgar 5 min	−0.563	0.258	4.759	0.029	0.570	0.344–0.944
	Renal dysfunction	2.073	0.697	8.840	0.003	7.947	2.027–31.164
Cerebral palsy	Lactate	0.201	0.073	7.665	0.006	1.223	1.060–1.410
	Any organ dysfunction	2.672	0.731	13.375	<0.001	14.475	3.456–60.619
Motor impairment	Lactate	0.173	0.075	5.375	0.020	1.189	1.027–1.377
	Cardiovascular dysfunction	2.179	0.749	8.460	0.004	8.837	2.035–38.369
Developmental delay	Lactate	0.222	0.081	7.535	0.006	1.248	1.066–1.463
	Apgar 10 min	−0.513	0.255	4.065	0.044	0.599	0.363–0.986
Epilepsy	Lactate	0.133	0.066	4.131	0.042	1.142	1.005–1.299
	Apgar 10 min	−0.558	0.233	5.753	0.016	0.572	0.363–0.903
Feeding impairment	Metabolic dysfunction	3.232	0.825	15.367	<0.001	25.333	5.033–127.503

Binary logistic regression analyses were performed using backward stepwise Wald elimination method. Variables with *p* < 0.10 in univariate analyses were included in the initial models. Lactate levels were independently associated with all adverse neurodevelopmental outcomes. Lower Apgar scores and presence of any organ dysfunction were also significant predictors. SE: standard error; CI: confidence interval.

## Data Availability

The data presented in this study are available from the corresponding author upon reasonable request due to privacy and ethical restrictions.
